# Longitudinal IQ Trends in Children Diagnosed with Emotional Disturbance: An Analysis of Historical Data

**DOI:** 10.3390/jintelligence6040045

**Published:** 2018-10-08

**Authors:** Tomoe Kanaya, Stephen J. Ceci

**Affiliations:** 1 Department of Psychology, Claremont McKenna College, Claremont, CA 91711, USA; 2 Department of Human Development, Cornell University, Ithaca, NY 14853, USA; sjc9@cornell.edu

**Keywords:** IQ, Flynn effect, Emotional Disturbance, historical analysis, longitudinal methods

## Abstract

The overwhelming majority of the research on the historical impact of IQ in special education has focused on children with cognitive disorders. Far less is known about its role for students with emotional concerns, including Emotional Disturbance (ED). To address this gap, the current study examined IQ trends in ED children who were repeatedly tested on various combinations of the WISC, WISC-R, and WISC-III using a geographically diverse, longitudinal database of special education evaluation records. Findings on test/re-test data revealed that ED children experienced IQ trends that were consistent with previous research on the Flynn effect in the general population. Unlike findings associated with test/re-test data for children diagnosed with cognitive disorders, however, ED re-diagnoses were unaffected by these trends. Specifically, ED children’s declining IQ scores when retested on newer norms did not result in changes in their ED diagnosis. The implications of this unexpected finding are discussed within the broader context of intelligence testing and special education policies.

## 1. Introduction

Regulations outlined in the Individuals for Disabilities Education Act [1] stipulate that children who are in need of special education services are required to undergo an IQ test as part of their qualification process. Further, if they are eligible for services, children must be re-tested at least every 3 years. Because of these IDEA stipulations, children with disabilities are one of the most heavily tested populations within America. The mandatory re-evaluations make it important for educators to understand the Flynn effect, the gradual rise in IQ scores documented in at least thirty-four countries around the world [2]. In the United States, the magnitude of the Flynn effect is approximately 0.31 points per year, totaling over three IQ points per decade on the Wechsler IQ scales.

Because of the Flynn effect, an IQ norm will produce inflated means over time. As a result, the test companies publish new norms that reset the previously inflated mean back to 100 points. Therefore, the Flynn effect is most pronounced at the time of the introduction of a new norm, whereupon scores suddenly and dramatically drop [3,4]. More specifically, Kanaya et al. [3] found that children who scored one standard deviation below the mean of 100 IQ points on the WISC-R lost approximately 5 IQ points when they were re-tested on the WISC-III. This drop resulted in a significant increase in intellectual disability (ID) diagnoses at the time of re-testing because many of these children had previously obtained IQs in the 70–75 range; therefore, a 5-point drop when the newer WISC-III norm was introduced was frequently sufficient to change their diagnosis to ID. In contrast to the 5-point drop experienced by those who were re-tested on the newer WISC-III norms, children who were tested and re-tested on the same WISC-R norms experienced a small, statistically insignificant rise of less than one point. Furthermore, unlike their peers who were tested on the newer WISC-III norms, there was not a significant increase in their ID diagnosis. Similar results have also been found in children diagnosed with learning disabilities—LD [5,6,7]. This body of research demonstrates that the Flynn effect can result in significant changes in children’s ID and LD diagnoses, as well as their educational experience in the absence of true changes in their cognitive abilities; random factors such as the norm used to score a child’s IQ performance affects their diagnosis independent of their actual ability. 

In contrast to the well-documented magnitude and impact of the Flynn effect on LD and ID diagnoses, little is known about the magnitude and impact of the Flynn effect on children who are diagnosed with emotional and/or behavioral disorders (ED) [8]. Indeed, many researchers have purposefully eliminated individuals with emotional/behavioral disabilities when charting Flynn effects, e.g., [9]. ED, however, is the fourth most prevalent special education category, representing approximately 6 percent of the special education population [10]; when combined with comorbidities, it represents over 40% of placements [11]. Therefore, examining the role of the Flynn effect on this population is necessary not only for understanding trends in ED diagnoses, but also to gain a comprehensive understanding of the impact of IQ on IDEA.

There are, however, substantial methodological demands to conducting this research. While ED is prevalent within the special education population, it only represents approximately 1 percent of all public school students currently [10], and was even less prevalent during the WISC and WISC-R years. Research on the Flynn effect also requires longitudinal follow-ups in order to see if the IQ test-re-test patterns have an impact on re-diagnosis rates. As such, data must be gathered from multiple school districts over several years in order to obtain the necessary follow-up data. Once these data are collected, analyses must be conducted in order to determine: whether the Flynn effect exists among individuals diagnosed with ED, a population that has been excluded from previous Flynn effect studies, and the impact (if any) of the Flynn effect on school children receiving special education services for ED.

## 2. Methods

### 2.1. Procedure

IQ data from special education assessments conducted by school psychologists were collected from nine different school districts across the United States representing a diverse sample of geographical regions (Midwest, Southeast, West, South) and neighborhood types (rural, urban, suburban). These districts were chosen by distributing a brief survey regarding the special education testing and archival process to the administrative personnel in every school district in the 48 continental United States and DC during 1998–2001. Of the approximately 300 responses, an overwhelming majority reported that they destroy their students’ records seven years after graduation. While approximately 20 districts reported archiving their special education records and granted us permission to examine their files, scheduling demands, data logistics (e.g., the archives were not physically accessible), and personnel changes (e.g., the principal who granted us permission relocated to a different district) made it impossible to collect data on all but nine of these districts. 

Data included students’ gender, age, testing date, IQ scores, test/re-testing norms used, and special education placement recommendations. Testing dates ranged from 1968–1999. If children were tested multiple times, all IQ test data available in the children’s files were collected, including test data from before and after the target test dates. Data were gathered by traveling to each school district and recording all necessary information from each student’s psychological testing file during 2000–2002. As a result of collecting longitudinal data on children who were tested during the targeted period, the dataset includes students who were tested once and did not qualify for services, as well as students who were repeatedly tested, typically for a required triennial reevaluation. Some children were repeatedly tested on the same test (e.g., repeatedly tested either on the WISC-R or on the WISC-III), and some were re-tested on a different test (e.g., initially tested on the WISC-R but re-tested on the WISC-III). Altogether, data from over 11,000 special education assessments were collected.

For the purposes of these analyses, children who were tested twice on the WISC norms and who were recommended for a diagnosis of ED on the WISC or WISC-R based on the first testing were extracted from the main dataset, which reduced the potential sample to 290 students. Of these, only 121 were re-evaluated at the same district, but it was necessary to include only the students who were tested on the WISC norms at the re-evaluation. These stringent selection requirements resulted in a sample of 109 school children.

### 2.2. Sample

Students were divided into 3 testing groups: (W-R) students who were initially tested on the WISC and re-tested on the WISC-R, (R-R) students who were tested and re-tested on the WISC-R, and (R-III) students who were initially tested on the WISC-R and re-tested on the WISC-III. The majority of these 109 children were male (*n* = 92, 84.4%; female *n* = 14, 12.8%; sex unknown *n* = 3, 2.8%). Unfortunately, there was little uniformity within the amount and type of demographic information provided in each student’s record. For example, most school districts did not document the race of the child, and socio-economic status or free-reduced price lunch status was not included in any district (most likely because the testing officials and practitioners would not have had access to such information). Further, while the Full Scale IQ scores were included in each student’s file, the Verbal and Performance scores were not. Table 1 lists the demographic information that was available for the total sample and each testing group.

## 3. Results

Difference scores (D-scores) between IQ at test 1 and test 2 were calculated for each group, as well as the average time between the two tests (in years). Testing dates ranged from 1968–1992 on the first testing and from 1975–1999 on the second testing. The test-retest correlation for the sample was statistically significant (*r* = 0.73, *p* < 0.001). A paired sample *t*-test revealed that the mean IQ at Time 1 (*M* = 85.94, *SD* = 14.04) and Time 2 (*M* = 84.82, *SD* = 12.96) were not significantly different (*t* (108) = 1.17, *ns*).

As can be seen in Table 2, the W-R, R-R and R-III groups replicated previous findings on the Flynn effect in school children tested for special education Specifically, there is a slight rise in IQ (*M* = 1.8, *SD* = 8.52) when tested and re-tested on the same norm, the R-R group. Thus, as has been found repeatedly, IQ scores tend to rise over time when the same norms are used by approximately 3 points per year. However, there is a sizable drop in IQ when ED children were re-tested on a newer norm—both the W-R (*M* = −3.8 points, *SD* = 11.35) and R-III (*M* = −7.64, *SD* = 9.98) groups. A multiple regression analysis was conducted for D-scores, where the W-R and R-III groups were dummy coded (the W-R group served as the baseline) and the number of years between tests was included as a covariate. Multiple regression analyses revealed the W-R and R-III groups experienced a significantly lower D-score compared to the R-R group after controlling for the amount of time between tests, *_adj_R*^2^ = 0.13, *F* (3, 107) = 6.31, *p* < 0.01 (see Table 3).

In order to determine if these IQ trends have an impact on ED diagnoses, the recommended diagnosis for each child at the second testing was tabulated and listed in Table 4. Chi-square analyses reveal that there were no significant differences in re-classification rates based on whether the child was re-tested on the same norm (the R-R) or re-tested on the different norm (the W-R and R-III groups), χ^2^ (1, *N* = 109) = 2.15, *ns*, indicating that the significant difference in D-scores present between these test-re-test groups did not have an impact on diagnosis at Time 2.

## 4. Discussion

The purpose of this study was to conduct a historical examination of the magnitude and impact of the Flynn effect on children diagnosed with ED under IDEA. By analyzing a sample of longitudinal IQ records obtained from special education evaluations from nine school districts around the country, the results reveal that ED children experience a substantial drop in IQ when they are initially tested on an older norm (i.e., the WISC or WISC-R) but re-tested on a newer norm (the WISC-R and WISC-III, respectively). Children who were tested on the WISC-R repeatedly, however, experienced a very small gain in scores upon retesting. This pattern is consistent with previous research on the Flynn effect with non-special education populations, which is marked by a slight rise in IQ as one norm ages, followed by a sharp decline upon the introduction of a newer norm. 

Our results suggest it is unlikely that the IQ decline experienced by individuals who are re-tested on a new norm are due to the inability for ED services to maintain the cognitive levels documented prior to the onset of ED services. Indeed, if this was the case, regression effects ought to have resulted in a slight positive trend given the low starting mean IQs of many ED students. It is important to note that our paired sample *t*-tests revealed no significant differences in IQ between the two tests. In other words, the IQ between the children in our sample appeared to be stable over time. It was not until the sample was divided into testing groups that significant differences, as predicted by the Flynn effect, were revealed. In addition, the Flynn effect is subtle on a year-by-year basis, and does not become pronounced until a new norm is published. This subtlety might explain why approximately one-third of directors of school psychology and counseling programs reported little to no familiarity with the Flynn effect as recently as ten years ago [12].

## 5. The Thin Lines between ED, LD and ID

Many studies, including student-level and system-level analyses, have demonstrated a negative impact of disruptive behavior on cognitive competence, e.g., [13,14]. Under IDEA, however, these special education categories are mutually exclusive, and since its inception as a diagnostic category, low-IQ children with ED are often bounced back and forth between ID and ED classes, a practice that has been observed for many decades (for an early example, see [15]). Along these lines, approximately 16% of our sample was diagnosed with LD or ID upon re-evaluation, supporting previous research findings that ED services may not be adequate in meeting the cognitive needs for all children who are diagnosed in this category, e.g., [11]. Interestingly, none of the children in our sample was diagnosed with “Multiple Disabilities”. To qualify for special education services under IDEA, children must display “concomitant impairments (such as intellectual disability-blindness or intellectual disability-orthopedic impairment), the combination of which causes such severe educational needs that they cannot be accommodated in special education programs solely for one of the impairments” [16]. While “deaf-blindness” is not an allowable combination, there is nothing preventing ID-ED or LD-ED as qualifying for the diagnosis. Given that the mean IQ in our sample was consistently low across testing combinations, most ED children would likely benefit from services that provide supplemental cognitive and academic resources in addition to emotional and behavioral management. Future research on IQ variations within the ED population and the “Multiple Disabilities” population at a national level would need to be conducted in order to make meaningful conclusions regarding these trends.

Contrary to previous findings on the impact of the Flynn effect on ID and LD diagnoses, however, the significant drop in IQ due to the use of the new WISC-R and WISC-III norms did not lead to a significant change in the number of children who qualified for ED diagnosis at the time of re-testing. Rather, approximately 60% percent of our total sample continued to be diagnosed as ED upon re-testing. Among those who were not diagnosed as ED upon re-testing, approximately one-third were placed in LD, and another one-third were placed out of special education entirely. In view of previous research showing that the Flynn effect has a large impact on ID and LD diagnoses, and given the co-morbidity of LD/ID with ED, it seems counterintuitive that the Flynn effect did not affect ED diagnoses in the present study. One possibility for this might be that the Flynn effect is having an impact on ED diagnoses in a less direct way than can be demonstrated by our data alone. A number of researchers have found wide variability among practitioners and policy makers regarding the exact criteria and method for diagnosing ED, a situation observed from the inception of special educational services [17,18]. Therefore, a child who meets the diagnostic criteria for ED and other categories in one school district may fail to do so in another.

## 6. Methodological Difficulties with Research on ED

While it is possible that a larger sample size may have revealed a significant change in diagnosis, our sample size is a reflection of how difficult it is to obtain longitudinal and historical IQ data on this population. Given the hypotheses, it was necessary to reduce the sample to those who took two WISC testings. As a result of this condition, less than half of the original sample of 290 students who received an ED diagnosis at the initial evaluation could be included in our analyses. Indeed, our original dataset contained over 11,000 special education testing records, but less than 1% of the sample fit our stringent criteria. The ED diagnosis, however, is not as prevalent as LD or ID, and many studies on this population have relied on comparable sample sizes, e.g., [11].

While we believe this strict criterion allows generalization regarding the Flynn effect that go beyond its specific boundaries, it is possible there are characteristics about students with ED that make it unlikely to be tested on the WISC norms multiple times throughout their lives. Future research is needed to determine the extent to which this criterion limits the generalizability of the findings to all ED students. If this is the case, then the role of IQ for ED diagnoses might need to be reconsidered in future conversations regarding IDEA. Furthermore, it is important to remember that the staffing demands needed to provide services to ED students are substantial, such as the need to hire behavior modification therapists, and the use of separate classrooms e.g., [19,20]. Therefore, even small changes to diagnosis rates could lead to significant financial and staffing costs for individual schools.

In view of the few longitudinal studies on ED children, the longitudinal and cross-sectional nature of our dataset provided a rare opportunity to examine IQ trends on the WISC norms over a span of multiple decades. Furthermore, a substantial proportion of the research on ED children is conducted in a clinical setting, where the special education diagnosis of ED can be confounded with a DSM diagnosis of an emotional or behavioral disorder, e.g., [20]. A common research technique within an educational setting is to interview school psychologists or to provide vignettes of school children displaying various symptoms in order to ascertain their likely diagnosis, e.g., [21]. While such studies provide valuable information, it is unclear if the data from them can be generalized to actual school evaluations. In contrast, the present study contains data from children from a geographically and socioeconomically diverse database of children around the country who were tested as part of actual special education evaluations. Thus, the results better lend themselves to be generalized to children who are evaluated for ED services than do studies that ask counselors to opine about diagnoses based on vignettes about fictitious students.

We did not have access to the behavioral or emotional data about each child, including any achievement test, psychiatric, and/or clinical data. The purpose of our study, however, was to examine the presence and impact of historical IQ trends within a population that has been excluded from previous studies on the Flynn effect, e.g., [2]. Accordingly, it would not have been appropriate to include such data in our analyses. Zimet, Zimet, Farley, and Adler [22], however, found no significant differences in IQ between ED children with a psychiatric disorder and non-psychiatric ED children. Furthermore, due to the wide variability in the criteria to qualify for ED services within and between different schools [23], it is unclear if there is a single behavioral measure applicable to the entire ED population.

Similarly, achievement tests are not always included in the testing records. This is likely because achievement tests were not common during the WISC and early WISC-R years, and are not a required component for ED diagnoses. If a student is being evaluated due to emotional and behavioral concerns, a school psychologist may be unwilling to extend the length of the evaluation in order to obtain achievement data, especially if the student is not exhibiting a deficit within a specific academic subject area (e.g., math, spelling, reading, etc.). IQ, on the other hand, is required in all IDEA evaluations, making it a particularly important measure for analyses within all special education categories. Given the comorbidity between ED and LD, as well as the cognitive deficits that are closely tied to the behavioral symptoms associated with ED, requiring achievement test, medical, and/or home observational data may lead to more precise diagnoses and effective treatment plans. 

## 7. The Future of the Flynn Effect (and ED)

Given that recent studies are showing a reverse Flynn effect within the normative population in the United States, e.g., [24] and an almost twofold increase in ED diagnoses [25], examining IQ trends on more recent norms within the population could provide valuable information for IQ and education researchers. Finally, we restricted the present analyses to the WISC norms in order to remain consistent with previous studies on the Flynn effect research, which heavily focus on the WISC series [2]. Determining the impact of these other tests would also be beneficial for understanding the different ways in which IQ and other standardized, cognitive measures affect ED diagnoses and the magnitude of the Flynn effect on all individuals in the IQ distribution.

## Figures and Tables

**Table 1:** Descriptive summary of demographic data, Verbal IQs (VIQ) and Performance IQs (PIQ) available by testing group.

Group	N	SEX		RACE		VIQ 1		PIQ 1		VIQ 2		PIQ 2	
		Male	Female	Black	White	Mean *(SD)*	*n*	Mean *(SD)*	*n*	Mean *(SD)*	*n*	Mean *(SD)*	*n*
W-R *^a^*	15	13	2	2	6	84.54 (12.97)	13	88.62 (16.95)	13	91.20 (10.85)	10	89.36 (6.52)	11
R-R *^b^*	69	56	10	5	12	84.80 (11.93)	40	89.75 (12.70)	40	85.82 (12.05)	39	86.18 (11.06)	39
R-III *^c^*	25	23	2	4	5	87.65 (14.36)	17	87.00 (16.52)	17	92.89 (16.59)	18	90.67 (16.96)	18
Total	109	92	14	11	23	85.44 (12.61)	70	88.87 (14.34)	70	88.52 (13.46)	67	87.88 (12.36)	68

*^a^* Students who were initially tested on the WISC and re-tested on the WISC-R. *^b^* Students who were tested and re-tested on the WISC-R. *^c^* Students who were initially tested on the WISC-R and re-tested on the WISC-III.

**Table 2:** Means (and standard deviations) on the three testing groups.

Group	N	IQ Time 1	Age 1 in Months	IQ Time 2	Age 2 in Months	D-Score	Months between Tests
W-R *^a^*	15	86.53 (14.20)	109.67 (20.26)	82.73 (15.19)	160.93 (17.23)	−3.80 (11.35)	49.40 (19.66)
R-R *^b^*	69	83.62 (12.18)	112.56 (24.04)	85.43 (11.74)	149.91 (22.70)	1.81 (8.52)	37.17 (11.79)
R-III *^c^*	25	92.00 (17.16)	104.29 (22.62)	84.36 (15.06)	147.52 (24.67)	−7.64 (9.98)	44.04 (19.75)
Total	109	85.94 (14.04)	110.30 (23.28)	84.82 (12.96)	150.90 (22.70)	−1.13 (10.04)	40.40 (15.61)

*^a^* Students who were initially tested on the WISC and re-tested on the WISC-R. *^b^* Students who were tested and re-tested on the WISC-R. *^c^* Students who were initially tested on the WISC-R and re-tested on the WISC-III.

**Table 3:** Multiple regression analyses predicted D-score with R-R as baseline group.

Model	*B*	*SE B*	*β*
Intercept	0.659	2.563	
W-R *^a^*	−6.001	2.770 *	−0.208
R-III *^c^*	−9.378	2.260 ***	−0.392
Years b/n tests	0.362	0.722	0.048

*^a^* Students who were initially tested on the WISC and re-tested on the WISC-R; *^c^* Students who were initially tested on the WISC-R and re-tested on the WISC-III. *_adj_R*^2^ = 0.13, *F* (3, 107) = 6.31, * *p* < 0.05, *** *p* < 0.001.

**Table 4:** Recommended special education diagnosis upon second test for all 3 testing groups.

Group	N	ED	LD	ID	No Placement	Otherwise Health Impaired	Unknown
W-R *^a^*	15	7 (46.7%)	3 (20.0%)	2 (13.3%)	2 (13.3%)	0	1 (6.7%)
R-R *^b^*	69	46 (66.6%)	9 (13.0%)	1 (1.4%)	8 (11.6%)	0	5 (7.2%)
R-III *^c^*	25	14 (56.0%)	2 (8.0%)	1 (4.0 %)	5 (20.0%)	1 (4.0%)	2 (8.0%)
Total	109	67 (61.5%)	14 (12.8%)	4 (3.7%)	15 (13.8%)	1 (0.9%)	8 (7.3%)

*^a^* Students who were initially tested on the WISC and re-tested on the WISC-R. *^b^* Students who were tested and re-tested on the WISC-R. *^c^* Students who were initially tested on the WISC-R and re-tested on the WISC-III.

## References

[1] Public Law 108-446 (2004). The Individuals with Disabilities Act of 2004.

[2] Flynn J.R. (2017). Reflections about intelligence over thirty years. Perspect. Psychol. Sci..

[3] Kanaya T., Scullin M.H., Ceci S.J. (2003). The Flynn effect and U.S. policies: The impact of rising IQ scores on American society via mental retardation diagnoses. Am. Psychol..

[4] Kanaya T., Ceci S.J., Scullin M.H. (2003). The rise and fall of IQ in special ed: Historical trends and their implications. J. Sch. Psychol..

[5] Fitzgerald S., Gray N., Snowden R. (2007). A comparison of WAIS-R and WAIS-III in lower IQ range: Implications for learning disability diagnosis. J. Appl. Res. Intellect. Disabil..

[6] Kanaya T., Ceci S. (2012). The impact of the Flynn effect on LD diagnoses in special education. J. Learn. Disabil..

[7] Sanborn K.J., Truscott S.D., Phelps L. (2003). Does the Flynn effect differ by IQ level in samples of students classified as learning disabled?. J. Psychoeduc. Assess..

[8] Mattison R.E., Blader J.C. (2017). What affects academic functioning in secondary special education students with serious emotional and/or behavioral problems?. Behav. Disord..

[9] Flynn J.R. (1987). Massive IQ gains in 14 nations: What IQ tests really measure. Psychol. Bull..

[10] The Condition of Education 2015. https://nces.ed.gov/pubs2015/2015144.pdf.

[11] Mattison R. (2015). Comparison of students with emotional and/or behavioral disorders as classified by their school districts. Behav. Disord..

[12] Hagan L.D., Drogin E.Y., Guilmette T.J. (2008). Adjusting IQ scores for the Flynn effect: Consistent with the standard of practice?. Prof. Psychol. Res. Pract..

[13] Rindermann H., Ceci S.J. (2009). Educational policy and country outcomes in international cognitive competence studies. Perspect. Psychol. Sci..

[14] Garwood J.D. (2018). Literacy interventions for secondary students formally identified with Emotional and Behavioral Disorders: Trends and gaps in the research. J. Behav. Educ..

[15] Reiss S., Levitan G.W., McNally R.J. (1982). Emotionally disturbed mentally retarded people: An underserved population. Am. Psychol..

[16] Sec. 300.8 Child with a disability. https://sites.ed.gov/idea/regs/b/a/300.8..

[17] Hanchon T., Allen R.A. (2013). Identifying students with emotional disturbance: School psychologists’ practices and perceptions. Psychol. Sch..

[18] Algozzine B. (2017). Toward an acceptable definition of Emotional Disturbance: Waiting for the change. Behav. Disord..

[19] Dunlap G., Kern L., Deperczel M., Clarke S., Wilson D., Childs K.E., White R., Falk G.D. (2018). Republication of “Functional Analysis of Classroom Variables for Students with Emotional and Behavioral Disorders”. Behav. Disord..

[20] Wiley A.L., Siperstein G.N., Forness S.R., Brigham F.J. (2010). School context and the problem behavior and social skills of students with Emotional Disturbance. J. Child Fam. Stud..

[21] Zabel R., Peterson R., Smith C., White M. (1982). Availability and usefulness of assessment information for emotionally disabled students. Sch. Psychol. Rev..

[22] Zimet G.D., Zimet S.G., Farley G.K., Adler S.S., Zimmerman T. (1994). Intellectual competence of children who are beginning inpatient and day psychiatric treatment. J. Clin. Psychol..

[23] Hanchon T.A., Allen R.A. (2018). The identification of students with Emotional Disturbance: Moving the field toward responsible assessment practices. Psychol. Sch..

[24] Rindermann H., Becker D., Coyle T.R. (2017). Survey of expert opinion on intelligence: The FLynn effect and the future of intelligence. Personal. Individ. Differ..

[25] McLeskey J., Landers E., Williamson P., Hoppey D. (2012). Are we moving toward educating students with disabilities in less restrictive settings?. J. Spec. Educ..

